# Supplementary material for: Probabilistic causal reasoning under time pressure
Source: PLoS One. 2024 Apr 11;19(4):e0297011. doi: 10.1371/journal.pone.0297011 (PMC11008876; doi:10.1371/journal.pone.0297011)
Supplement: S1 Appendix — (DOCX) [file pone.0297011.s001.docx]

# S1 Appendix: Analysis excluded participants

In both Experiment 1 and 2 we had a substantial dropout rate due to the a priori exclusion criterion we used on the overall response error. The cutoff point for error was 18%, meaning that all excluded participants performed worse than someone who would simply respond to each inference with ‘50%’ (see main text). This likely means that these participants either did not comply with task instructions or did not understand the task.

As we had decided upon the exclusion criterion before seeing the data, we here provide an additional analysis of the 35 excluded participants from both experiments to see whether they display similar behavior and whether their exclusion from the main analysis was justified. Specifically, we will first compare important overall response characteristics of the included and excluded participants. And secondly, we will redo the main analyses from the manuscript using the excluded participants.

## General response characteristics

First, we take a look at general response characteristics of the excluded participants. In addition to excluding participants, three types of responses were not included in the main analysis: invalid responses (where participants moved the joystick outside of the 0-100% range), missed responses (where participants did not respond before the deadline), and very fast responses (responses with an RT below 1.5 seconds). Each of these type of responses can indicate non-compliance or a lack of understanding of the task. Table S1 displays the percentage of these types of responses for the excluded and included participants. From these proportions we can see that the excluded participants exhibit more responses indicating non-compliance. Especially the 15% of responses that had an RT below 1.5 seconds is striking.

| **Table S1** *Percentage of non-compliant responses and overall response times* | | | | | |
| --- | --- | --- | --- | --- | --- |
|  | Invalid responses | Missed responses | RT < 1.5s | Mean RT | *SD* RT |
| Included participants | 0.4% | 0.2% | 2% | 4.40s | 2.46 |
| Excluded participants | 0.8% | 0.6% | 15% | 3.76s | 2.46 |
| *Note*. Percentage of responses that fall into one of three categories of responses indicating non-compliance, separately for participants included and excluded from the main analysis. Invalid responses refer to trials in which participants moved the joystick (see Methods) significantly outside the 0-100% range. Missed responses are trials on which participants did not respond before the deadlines. RT < 1.5s refer to trials with a response time under 1.5s. | | | | | |

Next, we look at overall response distributions. Figure S1 plots the overall response distributions of excluded and included participants per inference.


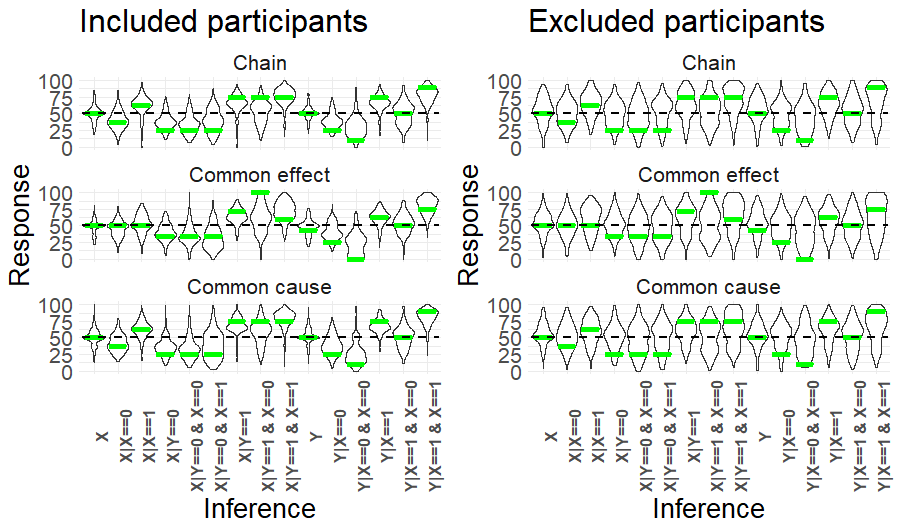


**Fig S2. Response distributions of included and excluded participants**

Violin plots of the distributions of responses of included and excluded participants for each inference separated by causal structure. Green dashes indicate the correct response.

From Figure S2 we can see that excluded participants have a considerably larger spread in responses for every single inference than the included participants. Moreover, the response distributions of excluded participants seem a lot more similar over the different inference types indicating that these participants responded similarly regardless of what inference they were asked to make. These are indications that these participants did not comply with task instructions or did not understand the task.

## Main analysis

Here we apply the same analysis as in the main text to the group of excluded participants. For details considering these analyses we refer the reader to the main text. For the sake of brevity we focus here on the main results regarding the effects of time pressure and confidence.

To test whether the time pressure manipulation impacted response times we regressed the Deadline factor on RTs and found that the effect of Deadline is indeed significant (*F*(2, 7145) = 624, *p* < .001; mean RT DL6 = 3.38s (*SD* = 1.05), DL9 4.07s (*SD* = 1.61), and DL20 5.31s (*SD* = 3.22)), similar as for the analysis in the main text.

Next, we investigated the overall SAT, that is, the influence of RT and deadlines on overall error. Contrary to the participants included in the main analysis, for the excluded participants we find no effect of deadline (χ2(2) = 1.62, *p* = .45), RT (χ2(1) = 0.0056, *p* = .94), nor their interaction (χ2(2) = 0.81, *p* = .67). This indicates that there was no effect of the passing of time nor of the deadline conditions on excluded participants’ response error.

Next, we look at the effect of time pressure on Markov violations and explaining away. We find the same pattern of results as with the participants included in the main analysis, namely that neither RT nor the deadlines affect Markov violations and explaining away. Specifically, for the common cause and chain structures we find no effect of RT (*F*(2, 2099) = 0.124 , *p* = .883, *BF*_01_ = 62.6) and Deadline (*F*(4, 2095) = 0.599, *p* = .664, *BF*_01_ = 235) on Markov violations, nor do we find such effects for the common effect structure (RT: *F*(2, 486) = 0.574 , *p* = .563, *BF*_01_ = 18.8; Deadline: *F*(4, 480) = 0.670 , *p* = .613, *BF*_01_ = 35.8). The findings are similar for explaining away, no effect of RT (*F*(2, 482) = 2.08 , *p* = .126, *BF*_01_ = 3.82) nor Deadline (*F*(2, 478) = 0.479 , *p* = .751, *BF*_01_ = 47.0).

The next analysis concerns conservatism. We find a significant main effects of RT (*F*(1, 2219) = 9.39 , *p* = .0022, *BF*_10_ = 1.28) on conservative responding, and we find mixed evidence for a main effect of Deadline (*F*(2, 2214) = 3.82 , *p* = .0221, *BF*_10_ = 0.468) as well as for the interaction effect of RT and Deadline (*F*(2, 2220) = 3.98 , *p* = .0187, *BF*_10_ = 0.427). This is similar as the results in the main text, where find a significant effect of RT for both experiments, but the evidence for the interaction effect is not as strong for the online experiment.

Lastly, we look at the role of confidence. We find that confidence has no effect on overall error (χ2(1) = 1.33, *p* = .249). This is contrary to what we found for the participants included in the main analysis for who where more confident in more accurate responses. That confidence is not associated with accuracy for the excluded participants could have three explanations. First, it could be that confidence is not a good index of participant’s uncertainty regarding their inference. Second, it could be that these participants were simply as confident in each of their responses, i.e. it could be that all their responses are just guesses. Thirdly, it could be that that these excluded participants did not report their confidence faithfully. The first of these reasons seems unlikely, as for the included participants confidence *was* a good index of their certainty regarding their inference. Both the second and third reasons indicate non-compliance and seem more likely.

For the participants included in the main analysis overall confidence was not associated with either Markov violations nor with explaining away, but it was associated with conservatism. For the excluded participants we find that confidence was not associated with Markov violations in common cause and chain structures (*F*(2, 1137) = 0.125 , *p* = .0883, *BF*_10_ = 0.011), we find some evidence for a relation of confidence with Markov violations in the common effect structure (*F*(2, 258) = 4.78 , *p* = .0091, *BF*_10_ = 4.56), and evidence for a role of confidence in explaining away *F*(2, 247) = 4.12 , *p* = .017, *BF*_10_ = 3.96). These results are different from the ones in the main text, where we did not find these effects. What is similar to the results in the main text is that the excluded participants are also less confident when they are more conservative (*F*(1, 1187) = 82.2 , *p* < .0001, *BF*_10_ > 1000).

In summary, there are some remarkable differences in the main results for the included and excluded groups of participants. First, that for the excluded participants there is no clear evidence for an overall SAT, i.e. the passing of time nor the deadline conditions significantly affected overall error. Moreover, for the excluded participants confidence is also not associated with overall error. Both these findings lead us to doubt whether these participants were motivated, complied with task instructions, and/or understood the task. There were also some small differences with regard to the relationship of confidence to Markov violations, but we refrain from interpreting these here as the fact that confidence is not associated with overall accuracy sheds doubt on interpreting confidence ratings as indications of participant uncertainty regarding an inference.

Taking these results together with our above findings relating to general response characteristics, we conclude that the group of excluded participants behaves substantially different from the group of included participants. This corroborates our doubt regarding whether the excluded participants were motivated, whether they complied with task instructions, or whether they understood the task. Based on these findings we judge that using the exclusion criterion of 18% overall error, which we determined before seeing the data, was justified.
