# Supplementary material for: Probabilistic causal reasoning under time pressure
Source: PLoS One. 2024 Apr 11;19(4):e0297011. doi: 10.1371/journal.pone.0297011 (PMC11008876; doi:10.1371/journal.pone.0297011)
Supplement: S2 Appendix — (DOCX) [file pone.0297011.s002.docx]

# S2 Appendix: Additional analyses experiment 2

We conducted the same regression analyses used for Experiment 1 for Experiment 2 as well. In the main text only the main results of these analyses relating to the reasoning errors are presented. The other results for Experiment 2 are concisely presented here, following the same structure as for Experiment 1 in the main text. For more information about the regression analyses see Experiment 1 in the main text.

### Manipulation check

To test whether the time pressure manipulation impacted response times we regressed the Deadline factor on RTs, and we found that the effect of Deadline is significant (*F*(2, 3999) = 247, *p* < .001, BF_10_ > 100).

### Overall SAT

Next we investigated the overall SAT, that is, the influence of RTs and time pressure on overall accuracy. We found a significant main effect of Deadline (χ2(2) = 11.3, *p* = .004), indicating a macro-SAT. Participants were more accurate when there was less time pressure. Post-hoc contrasts revealed that this is due to participants being significantly more accurate in the DL20 condition (*M* = 13.2, *SE* = 0.924) than in the DL6 condition (*M* = 14.7, *SE* = 1.04, *z*_DL6-DL20_ = 3.35, *p* = 0.002). Accuracy in the DL9 condition does not significantly differ from the other conditions (*M* = 13.8, *SE* = 0.968, *z*_DL6-DL9_ = 1.84, *p* = 0.159, *z*_DL9-DL20_ = 1.70, *p = .*204). There was no significant interaction effect of RT and Deadline (χ2(2) = 1.99, *p = .*369), and the main effect of RT was just not significant (χ2(1) = 3.60, *p = .*058).

### SAT Markov independence and explaining away

#### Markov violations Common cause and Chain

We found a significant main effect of ScreenedOff (*F*(2, 1171) = 84.0, *p* < .001), indicating that participants did not screen off, and thus violated Markov independence. The interactions of ScreenedOff with Deadline (*F*(4, 1171) = .959 , *p* = .429, BF_01_ = 48.8) and RT (*F*(2, 1177) = 2.73, *p* = .065, BF_01_ = 3.13) were both not significant, indicating that the violations of Markov independence were not impacted by time pressure nor response times. We did find a significant interaction between ScreenedOff and MidVar (*F*(2, 1171) = 72.2, *p* < .001, BF_10_ > 100)), indicating that the violations of Markov dependence were larger when the middle variable was present than it was not.

#### Markov violations Common effect

We again only found a significant main effect of ScreenedOff (*F*(2, 262) = 8.69, *p* < .001, BF_10_ > 100), indicating that participants violated Markov independence here. The interactions with ScreenedOff were not significant for both Deadline (*F*(4, 262) = 1.08, *p* = .368, BF_01_ = 9.93) and RT (*F*(2, 265) = 1.73, *p* = .180, BF_01_ = 3.89), indicating that there are no time pressure effects.

#### Failures to explain away

We found a significant main effect of AwayVar (*F*(2, 271) = 498, *p* < .001, BF_10_ > 100), indicating that participants did not engage in the normative explaining away pattern. The effect of knowing that other cause was absent is -4.88% (*SE* = 3.30), which is far from the CBN prediction, which says that the probability should increase by 28.6% compared to when the state of the other cause is unknown. The effect of knowing that it is present is +6.22% (*SE* = 3.38), which again is far from the CBN prediction of -11.4%.

There was no influence of deadlines on how participants explained away (*F*(4, 271) = 1.18, *p* = .318, BF_01_ = 11.7). However, we did find some evidence of an interaction of AwayVar with RT (*F*(2, 276) = 8.22, *p* < .001, BF_10_ = 0.986), as we found in Experiment 1 (see results in main text).

### SAT conservative inferences

Participants tended to respond conservatively, moving on average 5.0% (*SE* = 1.10, *t* = 4.56, *p* < 0.001) towards 50% from the normative response.

We found mixed evidence of an interaction of Deadline and RTs on conservative responding (*F*(2,1676) = 4.55, *p* = .011, *BF*_10_ = 0.739). Focusing on main effects, we find that there is no effect of Deadline on conservatism (*F*(2,1673) = 1.93, *p* = .15, *BF*_01_ = 21.3), but we find a large effect of RT (*F*(1,1681) = 21.5, *p* < .001, *BF*_10_ > 100) indicating that conservatism is sensitive to internal time pressure. Using post-hoc contrasts, we found that the effect of RT is significant in the 6s (β = 2.18, *SE* = 0.622, *t*(1678) = 3.51, *p* < .001) and 9s deadlines (β = 1.80, *SE* = 0.454, *t*(1675) = 3.96, *p* < 0.001), but not for the 20s deadline (β = 0.549, *SE* = 0.293, *t*(1678) = 1.84, *p* = .066). Pairwise contrasts revealed that the effects in the 6s and 9s conditions are not significantly different (*t*(1675) = 0.502, *p* = . 87), while they were different from the 20s condition (versus 6s: *t*(1678) = 2.40, *p* = .044; versus 9s: *t*(1675) = 2.35, *p* = .049). Hence there seemed to be a micro-SAT for conservative inferences in the 6s and 9s conditions, but not in the 20s condition.
