# Supplementary material for: Probabilistic causal reasoning under time pressure
Source: PLoS One. 2024 Apr 11;19(4):e0297011. doi: 10.1371/journal.pone.0297011 (PMC11008876; doi:10.1371/journal.pone.0297011)
Supplement: Attachment — Submitted filename: review.pdf [file pone.0297011.s003.pdf]

In this manuscript, the authors have conducted two probabilistic causal inference experiments by manipulating time pressure. The authors found the accuracy-tradeoff pattern in the behavior, as expected, and more conservative responses in the presence of time pressure. However, they found that Markov violations and failures to explain away, used as two other measures of reasoning error, stayed the same across the two experiments. The authors suggest that confidence plays an important role in dissociating the effects and that the reasoning error should be attributed to multiple processes.
The experimental design, analyses, and findings are interesting. However, so many analyses reported in the manuscript can distract from the main findings. Therefore, focusing more on the main results and moving auxiliary analyses to the supplementary materials can help the reader to understand the take-home message more accessible from the Results section.

Minor comments:

1) In Experiment 1, what is the logic behind the exclusion criteria of 18%? Why this specific number?
2) Have the authors performed power analyses to find the number of participants (41 in Experiment 1)? If not, post hoc power analysis can be helpful.
3) It is highly recommended to report effect sizes wherever applicable to give a better sense of the significance of the difference alongside the p-value.
4) The motivation and approach could be clearer in lines 378-385. It also needs to be clear how the CBN predictions are calculated in line 381. In general, more clarification on how the CBN predictions are obtained will be helpful.
5) In Figures 3 and 4 (and other applicable figures), showing the data points (instead of only the bar plot) will also be helpful.
6) It needs to be made clear where the results in lines 388-391 are coming from or how they have been calculated.
7) Line 446 needs to be clarified what the independent variable is.
8) Line 507 needs to be revised (incomplete sentence in the middle of the line).
9) In Experiment 2, since there is a pronounced unbalance in the gender of the participants, it will be helpful to recruit more female participants. Furthermore, in Line 518, does it mean 20/37 participants were excluded, and the results are based on 17 participants?
