# Supplementary material for: Probabilistic causal reasoning under time pressure
Source: PLoS One. 2024 Apr 11;19(4):e0297011. doi: 10.1371/journal.pone.0297011 (PMC11008876; doi:10.1371/journal.pone.0297011)
Supplement: Attachment — Submitted filename: Response to reviewers PO_2.docx [file pone.0297011.s004.docx]

Dear editor, reviewers,

We thank the reviewers for their kind remarks and encouraging comments, these helped us improve the manuscript considerably. We were happy to read that the reviewers generally regarded the work as interesting and valuable. In the remainder of this letter we will address the reviewers’ comments one by one.

Editor:

*Thank you for submitting your manuscript to PLOS ONE. After careful consideration, we feel that it has merit but does not fully meet PLOS ONE’s publication criteria as it currently stands. Therefore, we invite you to submit a revised version of the manuscript that addresses the points raised during the review process.*

*Specifically, Reviewer # 2 raises important concerns about the exclusion criteria, fast saccades, and interpretation of results in terms of the time to deadline vs. decision time. For the first concern, I suggest that you analyze data from the excluded subjects to test if they show any specific patterns or whether their behavior reflect poor performance due to certain factors (e.g., lack of motivation, misunderstanding the task, etc.). For the latter concerns, you could perhaps look at the data from the point of decision time to test whether longer deliberation time could have positive effect on the performance. Such analyses could provide a complementary account of the results, followed by appropriate discussion. The reviewers point out other concerns and suggestions that need to be addresses as well.*

To address the first concern, we have analyzed the data from the excluded participants as suggested. While our analysis is not able to discern what precise factors (e.g. a lack of motivation or understanding) leads to the behavior of these participants, it clearly indicates that the excluded participants behave different than the ones included in the main analysis. Moreover, the differences we find between the included and excluded participants do suggest a form of non-compliance or a lack of understanding for the latter. We have placed these analyses in the supplementary materials (S1 Appendix). We chose to not put them in the main manuscript as we do not want to distract from the (many) other findings and because we cannot be sure that these excluded participants engaged in causal reasoning, which is our main interest.

With regard to the issue of time to deadline vs. decision time, the statistical analyses we conducted and presented in the manuscript are able to detect both of those effects. Both these two effects describe a relationship between response times and responses (either raw responses or error). The perspective of time to deadline would predict responses to become less accurate with response times due to increasing time pressure, while the perspective of decision time would predict that as participants have more time to deliberate, their accuracy increases. Our results clearly point towards the former: responses become less accurate with increasing RTs. Hence, in discussing our results we mainly took the perspective of time to deadline. However, the perspective of deliberation is equally valid, even though the derived predictions are not borne out. To make sure that this is clear for the reader we have now added, in the results sections and General Discussion, a discussion of the perspective of deliberation time in relation to our results and model predictions. For more on this please see our response to Reviewer #2 below.

Reviewer #1:

*In this manuscript, the authors have conducted two probabilistic causal inference experiments by manipulating time pressure. The authors found the accuracy-tradeoff pattern in the behavior, as expected, and more conservative responses in the presence of time pressure. However, they found that Markov violations and failures to explain away, used as two other measures of reasoning error, stayed the same across the two experiments. The authors suggest that confidence plays an important role in dissociating the effects and that the reasoning error should be attributed to multiple processes.*

*The experimental design, analyses, and findings are interesting. However, so many analyses reported in the manuscript can distract from the main findings. Therefore, focusing more on the main results and moving auxiliary analyses to the supplementary materials can help the reader to understand the take-home message more accessible from the Results section.*

We thank the reviewer for this comment and agree that the complexity of our experiment and analyses forms a challenge to keeping the results readable. The many different conditions (3 deadlines x 3 structures x 27 inferences) require a somewhat elaborate results section. There is a trade-off between readability on the one hand and on the other hand being thorough and presenting sufficient results such that the interested reader can precisely follow our analyses. This, for example, is also related to the reviewer’s comment regarding effect sizes. We feel the need to report sufficient statistics, estimates, and post-hoc contrasts so that the reader can accurately gauge the size and robustness of our findings. Hence, we feel that the statistics we present are necessary and should not be removed from the main text, else it would imply that things are simpler than they actually are. However, we agree with the reviewer that the Results sections should be improved to be more accessible and readable. We have now worked on this by changing the structure and text of the results. With regard to the structure, we changed multiple headers to be more descriptive and created new subsections to delineate results regarding specific structures and effects (i.e. separating Markov violations in common cause/chain structures, Markov violations in common effect structure, and failures to explain away) such that each header now clearly specifies which results will be discussed. We have also added a sentence at the start of the results for experiment 1 describing how the results are structured. Additionally, we have sharpened the separate discussion sections for each experiment so that these give a clearer overview of the results.

*Minor comments:*

1) *In Experiment 1, what is the logic behind the exclusion criteria of 18%? Why this specific number?*

The reviewer rightly points out that our discussion of the exclusion criterion was lacking. The logic behind the exclusion criteria of 18% is as follows. If a participant simply responds with ‘50%’ on each trial, they will have a precision (which we now refer to as ‘error’ in response to another comment) of 18%. This is because the normative/correct responses are on average removed from 50% by 18 percentage points. Hence if a participant has an error lower than 18%, we can assume that on average they respond on the correct side of 50% (i.e. if the correct answer is higher (lower) than 50%, they tend to respond higher (lower) than 50%). This clearly indicates compliance and understanding of the task. We cannot be sure of compliance or understanding for a participant with an average error larger than 18%. Such a participant could be systematically responding on the wrong side of 50%, that is, responding higher (lower) than 50% when the correct response is below 50% and vice versa. We picked this theoretically motivated exclusion criterion before seeing the data (a priori) and ultimately kept it to ensure that the participants included in the analysis complied and understood the task to a reasonable degree.

We expanded the explanation of this criterion to include the above information in Section 2.1 where we introduce the criterion. In addition, we now provide an analysis of the excluded participant in the supplementary materials (S1 Appendix) which suggests that our exclusion of participants was justified.

*2) Have the authors performed power analyses to find the number of participants (41 in Experiment 1)? If not, post hoc power analysis can be helpful.*

No, we did not perform power analyses to find the number of participants prior to data collection. We were limited in terms of resources (time and money) which restricted the number of participants.

We decided not to run a post-hoc power analysis. Multiple reasons have been offered over the years for why the use of post-hoc power analyses are not as helpful as they might seem, making them a rather controversial method that has been argued to be conceptually problematic and uninformative (see e.g. Zhang et al., 2019; Heckman et al., 2022; Althouse, 2020; Goodman & Berlin, 1994), especially for highly parametrized models like the mixed regression models we use. A lot of these problems stem from the fact that the post hoc power analysis is based on a reinterpretation of the observed effects.

However, we understand the need to establish the validity and robustness of our findings. This is a main reason for why we ran the second experiment and had participants do almost 250 trials each. The fact that we have significant findings indicates that we had enough power to detect those effects. For the analyses in which we found null effects we have reported Bayes factors. Bayes factors express the amount of evidence for one hypothesis over another, and as such take sample sizes into account. In other words, if the sample sizes were too small, then we would have found Bayes factors to be consistently inconclusive.

Althouse, A. D. (2021). Post hoc power: not empowering, just misleading. *Journal of Surgical Research*, 259, A3-A6.

Goodman, S. N., & Berlin, J. A. (1994). The use of predicted confidence intervals when planning experiments and the misuse of power when interpreting results. *Annals of internal medicine*, 121(3), 200-206.

Heckman, M. G., Davis, J. M., & Crowson, C. S. (2022). Post hoc power calculations: an inappropriate method for interpreting the findings of a research study. *The Journal of Rheumatology*, 49(8), 867-870.

Hoenig, J. M., & Heisey, D. M. (2001). The abuse of power: the pervasive fallacy of power calculations for data analysis. *The American Statistician*, 55(1), 19-24.

Zhang, Y., Hedo, R., Rivera, A., Rull, R., Richardson, S., & Tu, X. M. (2019). Post hoc power analysis: is it an informative and meaningful analysis?. General psychiatry, 32(4).

*3) It is highly recommended to report effect sizes wherever applicable to give a better sense of the significance of the difference alongside the p-value.*

Unfortunately, there is no agreed upon method to compute standard effect sizes for individual model terms of mixed regression models (e.g. Rights & Sterba, 2019; Van Doorn et al., 2023) which is related to picking what partition of residual variance to use in computing the statistic (Singmann et al., 2021). Some methods exist for simpler experimental designs (e.g. Westfall, Kenny, & Judd, 2013), but not for designs with multiple factors and interactions as we use. We still used mixed regression models as they are numerous benefits over alternative approaches which makes the results from mixed models more likely to generalize (e.g. Barr et al., 2013; Judd, Westfall, & Kenny, 2012).

Where possible we report unstandardized effect sizes in line with general recommendations on reporting effect sizes (see Wilkinson, 1999; Pek & Flora, 2018, Singmann et al., 2021; Van Doorn et al., 2023). The idea behind unstandardized effect sizes is that the effect is expressed in terms of the response scale so that they are easy to interpret. This is what we do when we report post hoc contrasts. Our reporting of these post hoc contrasts either include the means or mean differences for conditions (for factors) or unstandardized regression coefficients (betas, for numerical independent variables), all in terms of the response scale. In addition we report the standard error for each these statistics.

For instance, in analyzing the overall SAT (Section 2.3.2), we report the mean and standard error of response error in every condition expressed in terms of the response scale (percentage points). This allows the reader to properly judge the effect size of the deadline conditions on error. We also report unstandardized regression slopes for the effect of RT separately for each deadline condition. For instance, for DL6 we report a mean slope for RT of 1.16. As this was a gamma regression we need to interpret these effects multiplicatively (see Section 2.2.3), meaning that for one SD increase in RT, precision was multiplied by 1.16, in other words, there is 16% more error.

In addition to reporting the relevant post hoc contrasts in terms of the response scale, we plotted all the important effects with the response scale (i.e. percentage points) on the Y-axis. This allows for easy visual inspection of effect sizes.

Refs:

Barr, D. J., Levy, R., Scheepers, C., & Tily, H. J. (2013). Random effects structure for confirmatory hypothesis testing: Keep it maximal. *Journal of memory and language*, 68(3), 255-278.

Judd, C. M., Westfall, J., & Kenny, D. A. (2012). Treating stimuli as a random factor in social psychology: a new and comprehensive solution to a pervasive but largely ignored problem. *Journal of personality and social psychology*, 103(1), 54.

Rights, J. D., & Sterba, S. K. (2019). Quantifying explained variance in multilevel models: An integrative framework for defining R-squared measures. *Psychological Methods*, 24(3), 309–338.

Pek, J., & Flora, D. B. (2018). Reporting effect sizes in original psychological research: A discussion and tutorial. *Psychological Methods*, 23(2), 208–225.

Singmann, H., Cox, G.E., Kellen, D., Chandramouli, S., Davis-Stober, C., & Dunn, J. C. (2021). Statistics in the service of science: Don’t let the tail wag the dog. *Computational Brain & Behavior*.

van Doorn, J., Haaf, J. M., Stefan, A. M., Wagenmakers, E. J., Cox, G. E., Davis-Stober, C. P., ... & Aust, F. (2023). Bayes factors for mixed models: A discussion. *Computational Brain & Behavior*, 6(1), 140-158.

Westfall, J., Kenny, D. A., & Judd, C. M. (2014). Statistical power and optimal design in experiments in which samples of participants respond to samples of stimuli. *Journal of Experimental Psychology: General*, 143(5), 2020.

Wilkinson, L. (1999). Statistical methods in psychology journals: Guidelines and explanations. *American psychologist*, 54(8), 594.

*4) The motivation and approach could be clearer in lines 378-385. It also needs to be clear how the CBN predictions are calculated in line 381. In general, more clarification on how the CBN predictions are obtained will be helpful.*

We thank the reviewer for pointing this out, the explanation of our analysis of explaining away should have been clearer as the analysis is inherently complex. We have improved this explanation and explicitly stated all the relevant calculations. (line numbers in revised manuscript: 399-408).

With regard to clarifying how the CBN predictions generally are obtained, we initially chose to leave out certain details to streamline the relevant sections. However, the reviewer is right in pointing this out as it would indeed have been better to specify such details. The CBN predictions, i.e., the correct probabilities, are computed by applying rules of probability calculus to the joint distribution of the causal network we taught to participants. The joint distribution is specified by the parametrization of the causal networks which participants learned by viewing samples as specified in Table 1. We have now added a sentence to where we introduce this parametrization (end of section 2.2.1) stating the above and referring the reader to a standard work on this topic.

*5) In Figures 3 and 4 (and other applicable figures), showing the data points (instead of only the bar plot) will also be helpful.*

We agree with the reviewer that plotting the data in addition to bars or regression lines can be very useful. We have added datapoints indicating the mean response time and mean error for Figures 3 and 4A respectively. We did not do this for any of the other plots, because with the amount of responses we gathered they would become very unclear (Figure 3 and 4A are the only ones where we plot means without overlapping conditions). E.g. with figure 4B, this would require the plotting of more than 2,000 datapoints per condition, i.e. more than 6,000 datapoints in each plot. Adding those points would not provide the reader with much additional information and it would result in main message of the figures being harder to read. To illustrate this, below we remade Figure 4B in the manuscript including data points.

*6) It needs to be made clear where the results in lines 388-391 are coming from or how they have been calculated.*

We thank the reviewer for pointing this out. Similar as in your minor comment (3), we should have provided more details on the relevant computations. We have now added such clarification, specifying how the mentioned percentages are computed. (Line numbers in revised manuscript: 410-417)

*7) Line 446 needs to be clarified what the independent variable is.*

Line 446 specifies ‘overall main effect of RT’, hence the independent variable is response times. It might have been that the word ‘overall’ has caused some confusion. We used the term ‘overall’ there to distinguish it from the interaction effects discussed before. However, this is superfluous as ‘main effect’ already denotes that it is a main effect, and so we removed the word ‘overall’. (New line number: 473)

*8) Line 507 needs to be revised (incomplete sentence in the middle of the line).*

Thank you for pointing this out. We revised the text such that the incomplete sentence is now a clause of the previous sentence. (New line number: 536)

*9) In Experiment 2, since there is a pronounced unbalance in the gender of the participants, it will be helpful to recruit more female participants. Furthermore, in Line 518, does it mean 20/37 participants were excluded, and the results are based on 17 participants?*

The gender unbalance is indeed pronounced, which is probably due to random sampling variability or the fact that males are overrepresented on the Prolific platform where we recruited participants. We do not have any hypothesis regarding gender differences and so we have no reason to expect that adding females would change any of the results.

Indeed, the results of Experiment 2 are based on 17 participants. This relates to your earlier comment on the exclusion criterion as well as comments by reviewer #2. The criterion is theoretically motivated and was chosen before viewing the data. This exclusion rate in Experiment 1 was a major reason for us to replicate our findings by conducting a second experiment. While the analysis indeed includes 17 participants, we still have sufficient datapoints as each participant provided 243 judgments. Moreover, we can be sure that they complied with task instructions.

We understand that the dropout rate is remarkable, even though it is not uncommon for complex reasoning studies such as these (see introduction to Experiment 2 in the main text). We now have added an analysis of the excluded participants in the supplementary materials (S1 Appendix). This analysis shows that there are multiple signs of non-compliance or a lack of understanding for this group and it shows that the excluded participants exhibit different response behavior overall.

Reviewer #2:

*The authors present an interesting set of experiments investigating the effect of time pressure on different kinds of errors in reasoning about causal scenarios. The investigation of time pressure on reasoning in general has many practical implications and the findings presented here would be a valuable contribution to the field. Although I think the manuscript should be published, I have a number of concerns and some minor suggestions.*

We thank the reviewer for their kind and encouraging comments.

*My main concern is regarding the large proportion of participants who were excluded from the analyses. In order for this research to be useful, one would have to be able to generalise the results to the general population and the large proportion of excluded participants makes this difficult. Although this has happened in other causal reasoning experiments, the generalisability issue remains nevertheless. I must admit that I didn’t fully understand how an average precision value of 18% corresponded to participants always responding with 50%, perhaps some further clarification might be worthwhile.*

The reviewer rightly points out that we did not explain the logic behind the exclusion criterion as clearly as we should have (Reviewer #1 also commented on this). We have now provided a more thorough explanation in Section 2.1, explaining that it has to do with the distribution of correct responses, which are on average removed from 50% by 18 percentage points. (In addition to Section 2.1, you can also find an explanation of this in our response to reviewer #1 minor comment 1).

We agree that generalizability is very important. This was a major reason for us to conduct a follow-up experiment, which allowed us to investigate how the findings would generalize to an online experiment. As we state at the end of section 3.4, this second experiment indicates that our findings are robust for the group of people we can be sure of that they understand the task and comply with task instructions. We understand that the proportion of excluded participants is large, but, as stated, this is not out of the ordinary for complex higher order reasoning tasks (see introduction to Experiment 2 in main text). Moreover, including participants in the main analysis who have clear signs of non-compliance or a lack of understanding would not improve the generalizability of our results regarding causal reasoning. This is due the fact that we have important reasons to think that the excluded participants were not displaying the same behavior as the included participants of whom we are confident that they performed as intended. To substantiate this, we have now provided an analysis of the excluded participants in the Supplementary materials (S1 Appendix) as suggested by the editor. Since our goal is to understand causal reasoning, generalizability would not be improved by analyzing data from participants who are likely not engaging in causal reasoning as intended.

*In any case, it would have been good to see the whole data, or at least data where a more lenient exclusion criterion was applied.*

Indeed, we agree that we could have provided more information regarding both the exclusion and the data of the excluded participants. We have now provided a supplementary analysis of the excluded participants as suggested by the editor in S1 Appendix. From this analysis it is clear that the excluded participants responded differently than the participants included in the main analysis. Moreover, it seems that the excluded participants were either not motivated to do the task to the best of their abilities or did not understand the task. We chose to not include an analysis of the whole data in the manuscript as it seems that the two groups of participants display different behaviors. Hence, what we will find with an analysis of the whole data would be somewhere in between of what we find for the included and excluded groups of participants.

*If I’m reading Fig 4 correctly, responses with very fast reaction times were most accurate. If so, wouldn’t this microSAT be the opposite to the macro one observed between conditions, and actually the opposite to a speed-accuracy trade-off altogether? Perhaps a brief discussion of this would be good even at that point in the manuscript, and I don’t think the effect shown in Fig 4b should be called an SAT effect since it is quite the opposite.*

*The authors also refer to conservatism (decisions closer to 50%) as a microSAT, but again, a speed accuracy trade-off implies more accurate decisions with longer reaction times, which is not what happened. Perhaps a different term than SAT should be used to describe these effects.*

The reviewer is reading Figure 4 correctly, the microSAT is in the opposite direction of the macroSAT. The term SAT is indeed sometimes used to refer to the relationship between increased reaction times and increased accuracy. However, in the literature on SATs, and specifically in the highly influential review article on SATs by Heitz (2014), the term SAT is used to refer the relationship between RTs and accuracy regardless of what direction this relationship is in (they refer to it as “the tendency for decision speed to covary with decision accuracy” Pg. 1). While the macroSAT tends to be in the increasing direction, the microSAT is known to vary in form and direction (see Pg. 9 and Figure 5 in Heitz, 2014). Based on this we decided to keep using the term to be in line with the existing literature on SATs. However, we understand the reviewer’s point, as with a ‘trade-off’ one would expect a particular direction of the relation such that one performance index (e.g. speed) can be traded off for another (accuracy). This is not the case here (as it can be for the microSAT), and so colloquially one might use the word ‘relationship’ or ‘balance’.

To make this issue clearer in the main text, we follow the reviewer’s suggestion and added a brief discussion of this point in the text near Figure 4 in addition to adding a sentence on this point to the discussion of the experiment (Section 2.4) where we also refer the reader to the Heitz paper.

Heitz, R. P. (2014). The speed-accuracy tradeoff: history, physiology, methodology, and behavior. *Frontiers in neuroscience*, 8, 150.

*If the authors were interested in decisions made closer to the deadline, I wonder if it would be useful to plot the time remaining (i.e., deadline – RT) rather than z-scored RTs, which makes interpreting the effects more difficult.*

*It would have been useful to plot the data points as well (though I understand those might be messy plots) and test for non-linear trends, as I suspect that very fast decisions and decisions closer to the deadline might both be more suboptimal than decisions with medium reaction times.*

We thank the reviewer for their suggestion. In an earlier phase we also thought of plotting the time to deadline. However, we found out that it does not provide extra information and it makes the plot harder to interpret because one has to think in ‘negative RT’. Plotting time to deadline instead of RT would do two things to our plots. Within deadline conditions it would simply flip along a vertical axis, that is, create a mirrored image of the original. Second, the grouping of conditions would overlap differently, as instead of the data from each condition being pegged at rt=0 (the minimum response time), they would be pegged at DL-rt=0 (the maximum response time). As an illustration, we redid figure 4B with time remaining on the X-axis. As we can see it provides the same information as the original figure.

We agree that plotting data points is generally useful, however as we have thousands of data points this would make our plots very messy and less readable indeed. We have provided an illustration of this in response to Reviewer #1 minor comment 5.

*The authors frame their RT results in terms of time pressure (i.e., deadline – RT, or time left to respond), but nevertheless, the closer the participant responds to the deadline, the more time they would have spent thinking about their answer. So one could argue that these RT effects are contrary to the time pressure hypothesis if one focuses on the length of the RT rather than time remaining to respond. I am not arguing that the time remaining does not influence the participants’ judgments, I am just pointing out that one could focus on either the length of the response versus the length of the remaining time before the deadline and focusing on these two time periods generates opposite predictions. This makes generating some clear predictions from the models discussed more difficult. For example, one could turn many of the arguments around by referring to ‘rushed responses’ (short RTs) rather than internal time pressure (long RTs closer to the deadline). Although the results suggest that people may have experienced some sort of deadline pressure, had anyone made predictions from these models before seeing these results they might have made opposite predictions.*

We thank the reviewer for raising this point as it is an important topic within the SAT literature (see Heitz, 2014). Within the SAT this is discussed in terms of slow versus quick errors. Whether the effect of deliberation time or that of increasing time pressure is more significant is an empirical question. This is why we did not have a specific hypothesis concerning the direction of the microSAT. As the reviewer mentions, our results clearly suggest that the effect of time pressure is more important for our task than the deliberation time within deadline conditions, as we find that accuracy decreases with RT. In this way the results justify our focus on the time remaining and it was these results that led us to frame our findings this way. However, as the reviewer points out, looking at deliberation time is a valid perspective as well. We had already discussed this perspective in light of the role of confidence. In section 3.3.2 we discuss that sometimes long RTs reflect more deliberation, which is associated with high confidence, and sometimes long RTs reflect the difficulty of the problem, which is associated with low confidence. In addition, we have now added a short discussion of this issue of deliberation time at the start of our general discussion (Section 4.1).

The models we discuss in the General Discussion actually predict what the reviewer is pointing out: Sampling theories predict an increase in accuracy with increased deliberation time as they can take more samples, and heuristic theories would predict that an increase in deliberation time would allow participants to rely less on simple heuristics and thus improve their accuracy. We have now made this point explicit in the sections where we discuss these models (Section 4.2.1 and 4.2.2).

*Minor comments:*

*I would suggest using the word ‘error’ rather than ‘precision’, since it better reflects what was measured (precision implies better performance at larger values, whereas error more accurately reflects deviance from a normative answer).*

We thank the reviewer for pointing this out. Throughout the development of this research we have discussed using multiple words for this, as ‘accuracy’ itself does not fit well. We agree with the suggestion that ‘error’ is more intuitive, and hence we have change ‘precision’ to ‘error’ or ‘response error’ throughout the manuscript.

*Perhaps the notation in the legend of Fig. 6 should follow the notation used in the previous figures (e.g., p(Xi|Y, Xj = 0) rather than non-queried cause absent).*

Using the probability notation would indeed be more consistent with the other figures. We have changed it according to your suggestion.

*The sentence ending with ‘…a probability that was in the oppositive half of the measurement scale as the normative response’ (page 20) was unclear to me, though the explanation that followed was clear.*

That sentence indeed could have been more clear, thank you for pointing this out. We have modified the sentence to be more clear.

We thank you again for reviewing our work and considering our work for publication.

Kind regards, on behalf of all co-authors,

Ivar Kolvoort
