# Supplementary material for: Probabilistic causal reasoning under time pressure
Source: PLoS One. 2024 Apr 11;19(4):e0297011. doi: 10.1371/journal.pone.0297011 (PMC11008876; doi:10.1371/journal.pone.0297011)
Supplement: Attachment — Submitted filename: Response to reviewers PO_revision2_1.docx [file pone.0297011.s005.docx]

Dear editor, reviewers,

We thank the reviewers and the editor for their kind remarks and helpful comments. We were happy to read that the reviewers feel that all (substantive) comments have been addressed and that only a few minor comments remain. In the remainder of this letter we will address the editor’s comment and the remaining comments by Reviewer #2.

Editor:

*Thank you for submitting your manuscript to PLOS ONE. After careful consideration, we feel that it has merit but does not fully meet PLOS ONE’s publication criteria as it currently stands. Therefore, we invite you to submit a revised version of the manuscript that addresses the points raised during the review process.*

*More specifically, Reviewer # 2 has a few minor concerns (see below) that need your attention and response.*

*In addition, it would useful to create a subsection with header for "Data Availability" (under Methods) to make it easier for the readers to find the data and analysis code. Please note that your response and revised manuscript will not be sent out to review again and only will be evaluated at the editorial level.*

We thank the editor for their help in overseeing this process and for helping to improve our manuscript. We have added a subsection “Data Availability” in the Methods section as suggested (section 2.2.4, pages 15-16). We address the minor comments by Reviewer #2 one by one below.

Reviewer #2

*I think the authors answered all my queries and made the relevant changes or explained why they chose not to. I only have a few minor comments.*

*The choice of 18% error as the cut-off to exclude participants was still difficult to understand, though I think I’m a little closer to understanding it. I presume it has something to do with the normative responses shown in Figure 5, if so, I think it should be clearer to the reader, one might even refer to the figure. And if I understood this criterion correctly, I agree that errors larger than 18% might mean that participants are systematically making judgments that are inconsistent with probabilistic reasoning.*

Indeed, the cut-off of 18% is related to the normative responses shown in Figure 5 (as well as to the normative responses indicated in other figures). The 18% refers to how far removed on average a participant’s responses were from the normative response, so it is indeed completely dependent on the normative responses. The normative responses are indicated in multiple figures, most clearly so in Figure 7, where the normative response for each inference is plotted as a green dash. We have taken up your suggestion to refer to a figure in the section where the cut-off is explained at the point where we indicate that the error is dependent on the distribution of normative responses (Section 2.1, line nr. 166). However, we refer to Figure 7, as it gives the reader an easy way to inspect the distribution of normative responses.

*The percentage of fast responses is quite high for the excluded participants, and I would also report the average reaction time or the distribution of RTs, since even RTs of 2-3 s might be quite fast for complex reasoning problems.*

Indeed, the percentage of fast responses is notably higher for the excluded participants, this is one of the indicators that these participants either did not comply with task instructions or did not understand the task. We agree with the reviewer that it would be insightful to present some more information concerning the response times, and hence we have, as suggested, added the mean and standard deviation of response times for the included and excluded participants to the table where the percentage of fast responses is mentioned, that is, in table S1 in S1 Appendix: Analysis excluded participants.

*I wasn’t sure what the authors meant by ‘…When participants failed to answer before the deadline, which happened on 19 trials in total, …’ Do they mean it happened on 19 test trials on average across participants?*

We thank the reviewer for pointing out the ambiguity in that sentence. “in total” in this sentence refers to all participants, that is, across all participants there were only 19 trials on which a response was not recorded before the deadline. We rephrased the sentence to clear this up, it now states “When participants failed to answer before the deadline, which happened on a total of 19 trials over all participants, …” (Line nr 274-275 in the revised manuscript).

*I still think SAT is a poor choice of terminology given the meaning of ‘trade-off’, but I understand that the authors chose to be consistent with previous work. The additional explanations in the results and discussion sections regarding the direction of the relationship between RT and accuracy were useful and should clear up any potential misunderstandings.*

We understand the reviewer’s concerns, as explained in our previous response we chose to be consistent with previous work, but we agree that the concept of a ‘trade-off’ is somewhat strained in how SAT is used in the literature. We are happy to hear that our additional explanations are useful and help to avoid potential misunderstandings.

*On a minor note, the scrambled figures were somewhat difficult to work with and I couldn’t find Figure 8 (though I found it in the original submission).*

Our apologies for not making it easy to find Figure 8. We do not exactly know what happened, but it indeed seems like Figure 8 was not included in the compiled PDF for the revision. The figure did not change in either this or the previous revision, but we will re-upload it to make sure it is available. Similarly, we apologize for the ‘scrambled figures’. It seems that in the compiled PDF of the revision the figures got ordered based on the order of uploading, and not based on figure number, which indeed makes it difficult to look up the right figure. We will reorder the figures in the submission portal.

We thank you again for reviewing our work, helping us to improve the manuscript, and for considering our work for publication.

Kind regards, on behalf of all co-authors,

Ivar Kolvoort
